# Adaptive Filtering for the Maternal Respiration Signal Attenuation in the Uterine Electromyogram

**DOI:** 10.3390/s22197638

**Published:** 2022-10-09

**Authors:** Daniela Martins, Arnaldo Batista, Helena Mouriño, Sara Russo, Filipa Esgalhado, Catarina R. Palma dos Reis, Fátima Serrano, Manuel Ortigueira

**Affiliations:** 1 NOVA School of Science and Technology, NOVA University Lisbon, 2829-516 Caparica, Portugal; 2 UNINOVA-CTS, NOVA School of Science and Technology, NOVA University Lisbon, 2829-516 Caparica, Portugal; 3 Faculty of Sciences, Lisbon University, Campo Grande, 1749-016 Lisbon, Portugal; 4 CEAUL Faculty of Sciences, Lisbon University, Campo Grande, 1749-016 Lisbon, Portugal; 5 Department of Obstetrics, University Central Hospital Lisbon (CHULC), 1169-050 Lisboa, Portugal; 6 Comprehensive Health Research Centre (CHRC), NOVA Medical School, Universidade NOVA de Lisboa, 1169-056 Lisboa, Portugal

**Keywords:** uterine electromyography, electrohystherography, alvarez waves, adaptive filters, pregnancy monitoring, respiratory electromyography

## Abstract

The electrohysterogram (EHG) is the uterine muscle electromyogram recorded at the abdominal surface of pregnant or non-pregnant woman. The maternal respiration electromyographic signal (MR-EMG) is one of the most relevant interferences present in an EHG. Alvarez (Alv) waves are components of the EHG that have been indicated as having the potential for preterm and term birth prediction. The MR-EMG component in the EHG represents an issue, regarding Alv wave application for pregnancy monitoring, for instance, in preterm birth prediction, a subject of great research interest. Therefore, the Alv waves denoising method should be designed to include the interference MR-EMG attenuation, without compromising the original waves. Adaptive filter properties make them suitable for this task. However, selecting the optimal adaptive filter and its parameters is an important task for the success of the filtering operation. In this work, an algorithm is presented for the automatic adaptive filter and parameter selection using synthetic data. The filter selection pool comprised sixteen candidates, from which, the Wiener, recursive least squares (RLS), householder recursive least squares (HRLS), and QR-decomposition recursive least squares (QRD-RLS) were the best performers. The optimized parameters were L = 2 (filter length) for all of them and λ = 1 (forgetting factor) for the last three. The developed optimization algorithm may be of interest to other applications. The optimized filters were applied to real data. The result was the attenuation of the MR-EMG in Alv waves power. For the Wiener filter, power reductions for quartile 1, median, and quartile 3 were found to be −16.74%, −20.32%, and −15.78%, respectively (*p*-value = 1.31 × 10^−12^).

## 1. Introduction

Premature birth, defined by the World Health Organization (WHO) as any birth occurring before 37 completed weeks of gestation [1], carries significant risks for both the mother’s and newborn’s health. According to reports provided by this entity, about 15 million premature births occur annually, of which, more than 1 million children die each year due to its complications. Those who survive often face impairments that include learning, visual, respiratory, and hearing deficiencies [2]. According to the same source, prematurity is the leading cause of newborn death in the world and second leading cause of death in all children. For all these reasons, the prediction of preterm births has been a topic that has attracted researchers’ attention. Some risk factors for premature birth, such as the previous history of preterm birth, ethnicity, low socio-economic status, maternal weight, smoking, and periodontal status, seem to play a minor role [3]. The majority of spontaneous preterm deliveries occur in women with no identifiable risk factors for this condition [4,5,6]. The pathophysiology of this condition is not completely understood [4].

To overcome these problems, recent studies have emerged [7,8,9,10] regarding the electrohysterogram (EHG) potential for applications in preterm and term birth prediction. The EHG, also called uterine electromyogram, consists of non-invasively recording the electrical signal generated by the contractile activity of the uterus [11]. Additionally, the analysis of this bioelectrical signal is useful for the general monitoring of uterine activity during pregnancy. The EHG also contributes to advances in uterine electrophysiology [8].

The EHG signal includes noise from the maternal respiration electromyogram (MR-EMG), motion artifacts, and maternal electrocardiogram (ECG), which affects the interpretation of the results. The MR-EMG signal present in the EHG is an outcome of the diaphragm and skeletal muscle movements associated with the chest wall [12,13,14]. The MR-EMG signal is a prevalent interference in the EHG, since its frequency band (from 0.20 to 0.34 Hz) [15,16] overlaps with the frequency band of one of the most important components composing the uterine signal, the Alvarez (Alv) waves, whose bandwidths range from 0.20 to 0.40 Hz [17]. According to this study, there are two subtypes of Alv waves: Alvarez low (AlvL) waves, with peak frequencies between 0.2 and 0.3 Hz, and Alvarez high (AlvH) waves, with peaks between 0.3 and 0.4 Hz. The correct identification and analysis of the Alv waves could be of paramount importance in the prediction of preterm delivery. When interpreted with the correct tools, EHG data incorporated into clinical algorithms could allow for better patient risk-stratification and avoidance of unnecessary hospitalizations and tocolytic therapy, both of which are not unharmful for patients.

Indeed, signal noise attenuation is an important issue in biomedical data analysis. In this context, the application of classical filtering techniques is one of the widely used options. However, when the bandwidth of the interference overlaps with the bandwidth of the signal of interest, classical filtering techniques produce limited results, since, by attenuating the noise, they also reduce the components of the signal of interest [18]. To overcome this effect, adaptive filtering techniques have been considered a valid alternative, since they may allow for the attenuation of the noise from the signal, while significantly preserving the characteristics of the latter, even if both exhibit an overlap in the frequency band. Another advantage of adaptive filters is the adaptation of their coefficients to the varying characteristics of the noise [19,20]. However, this technique requires a robust noise component estimation, i.e., the MR-EMG, in the study at hand. The respiration data used in this work were calculated indirectly by the electrocardiogram-derived respiration (EDR) algorithm applied to the maternal ECGs and retrieved from the EHGs of the considered database in this work.

The first report of an EHG recording is from Otto Bode in 1931 [21]. The technique has been evolving ever since, with contributions regarding signal processing and interpretation of the EHG signal [22,23,24,25,26,27] for pregnancy monitoring. With this perspective, the understanding of the EHG signal demands the study of its different components, including the Alv waves.

Alv waves were firstly described by Alvarez and Caldeyro in 1950 as high-frequency, low-amplitude waves reflecting myometrial contraction activity. The used signal was obtained through a pressure sensor, placed invasively in the vicinity of the uterus [28]. In recent times, Alv waves have become the subject of study, in the context of their possible contribution to pregnancy monitoring and term or preterm birth prediction, with an emphasis on the EHG as the used signal [8,17,29].

If, on the one hand, studies advocate that Alv waves can trigger the occurrence of more synchronous contractions, with increasing intensity leading to term or preterm labour [30,31,32,33]; on the other hand, other researchers support a non-causality relationship or are skeptical about it [34,35]. The latter group is, however, the minority [29]. Regardless of the methodology used to analyze Alv waves, a pre-processing noise attenuation step is required. Moreover, with Alv waves being low-amplitude events, noise contamination can be exceedingly detrimental for the results of the downstream algorithms to be applied.

Given this, a denoising step is required to be applied to the EHG and specifically designed to target the noise contamination that may affect Alv waves. Granted, this denoising process will also improve the EHG, in general. As mentioned earlier in this section, adaptive filters seem to be an appropriate technique for attenuating the MR-EMG from the EHG, a requirement for the reasons presented above. So, the scope of the herein presented work regards the application of adaptive filters for the task under study.

A survey was conducted focusing on the literature, concerning the adaptive filtering applications to the EHG. Upon literature inspection, it was considered advantageous to highlight the adaptive filtering publications in three categories, according to the following criteria:

- Retrieval of the fECG [36,37,38];
- Maternal ECG, and electronic electromagnetic interferences attenuation [20,39];
- Pre-term and term prediction and signal-to-noise ratio improvement in the EHG [19,40].

Regarding the retrieval of the fECG, the comments are as follows: in 2011, Liu et al. [36] presented an application of RLS and the normalized least mean squares (NLMS) adaptive filters to the EHG. The goal of this study was to extract the fetal ECG (fECG) from the collected abdominal signal, which contained both the mother’s and fetus’s ECG signals. It was concluded that the RLS algorithm is a signal processing technique suitable for fECG extraction. The parameters’ filter selection criteria were not disclosed, and neither was the population under study. The paper seems to investigate the adaptive filters’ capability to detect the fECG in the EHG, as a proof of concept. In 2013, Khalaf et al. [37] demonstrated that it was possible to obtain the fECG using the EHG. A combination of a blind source separation method with adaptive filtering (LMS) attenuated the noise arising from the maternal ECG. The parameter information was undisclosed. The studied population included five subjects. Blind source separation techniques were combined with adaptive filtering to attenuate the maternal ECG signal. Kahankova et al. [38] studied the application of the RLS and fast transversal filter (FTF) algorithms to the EHG signal to extract the fECG. The results demonstrated that the output of the RLS adaptive filter was identical to the reference signal, which supported the hypothesis that this algorithm would be advantageous for extracting the fECG from the EHG signal. A comparative analysis with previously used adaptive filtering algorithms was performed, which demonstrated that the standard RLS algorithm significantly outperforms the remaining tested methods. Synthetic data were used for parameter optimization using a grid of values. The authors suggest using real data in future works. These studies highlight the RLS filter as the best performer, except for Khalaf et al., where the LMS was used, along with independent components analysis (ICA) techniques. In this last study, a comparison between the LMS and other filter architectures seemed not to have been performed. Additionally, the authors reported a successful retrieval of the fECG and, consequently, adequate attenuation of the maternal ECG.

Regarding the maternal ECG and electronic electromagnetic interferences attenuation, the comments were as follows. In 2016, Limem et al. [20] looked into the efficiency of using the LMS and RLS algorithms for noise attenuation from the EHG signals, using the simulations performed via the MATLAB^®^ and Simulink^®^ platforms. The filter parameters were undisclosed. Four subjects were under study. The adaptive filter results were compared with the band-pass and wavelet filters. In a more recent study [39], in 2019, the same research group validated the previous results. Similar to the aforementioned studies, the RLS was the best performer for the task under study.

Concerning pre-term and term predictions and signal-to-noise ratio improvement in the EHG, the comments are as follows. J. Terrien et al. [19] used adaptive filters, namely least mean squares (LMS) and recursive least squares (RLS) to increase the signal-to-noise ratio in the monopolar EHG. Afterward, he compared the results with those using the Laplacian filter and concluded that the RLS filtering algorithm provides a higher signal-to-noise ratio than the LMS method or Laplacian filter. An accurate parameter selection is deemed to be a future work task, as well as the need to include a higher number of uterine contractions. This was a forerunner work, regarding EHG and adaptive filters. On the other hand, Smrdel et al. [40] used adaptive autoregression as one of the methods to estimate the spectrograms of the EHG signal. The goal was to separate and classify the delivery date as term or preterm. A set of filter parameters were tested. Frequency bands up to 4 Hz were under investigation. The study raises the possibility of pre-term delivery prediction from as early as the 23rd gestational week. These two studies highlight the RLS filter as a competent method to generally denoise the EHG; although, the second study did not include the exploration of other adaptive filter configurations.

Despite the following study not using the EHG, it is still a contribution to the pregnancy monitoring field. Cardiotocography was used, instead of the EHG. Huiling Tong et al., in 2017, presented a study regarding the application of adaptive filters to the intrapartum cardiotocography method for fetal monitoring [41]. Three adaptive filtering algorithms were used for the fetal monitoring system’s automated analysis. The proposed algorithms outperformed the classical filtering method for recognizing uterine contractions. It was observed that adaptive filtering provided the highest performance, compared with the two classical algorithms. A dedicated user interface was developed in Matlab^®^.

Table 1 provides detailed information about the aforementioned studies. Outside of the context of the EHG, adaptive filters have been used to attenuate noise in other types of biomedical signals, such as the ECG [42,43], shoulder and neck electromyogram [44], electroencephalogram [45], and electrogastrogram [46].

Despite the wide applicability of adaptive filters in the noise attenuation of biomedical signals, so far, to the best of the authors’ knowledge, the herein presented study is the first endeavour, regarding MR-EMG reduction in the EHG for improved Alv waves retrieval. The performance of adaptive filters, in turn, depends on the choice of their parameters at the time of implementation. This is one of the main challenges in the adaptive filtering context. Often, the selection of parameters is achieved based on previous studies. Thus, the propagation of parameter values that are not optimized for the application under study may occur. In the herein presented work, an automatic optimization process for adaptive filtering parameters’ selection is presented, regarding the application under study. This optimization process includes the selection of the best-performing adaptive filter architectures amongst a pool of 16 possibilities. This innovative approach can be used in other adaptive filter applications for noise attenuation, namely in the biomedical field. The next step is the application of the optimized filters to the real EHG signals.

As mentioned earlier, Alv waves have been pointed out as crucial components with the potential for term and pre-term birth prediction. If Alv waves are contaminated with maternal respiration, this may compromise its possible use as a tool to predict term or pre-term labour. Additionally, medical decision-making, regarding tocolytic therapy, whose side effects must be considered, can potentially benefit from Alv waves signal integrity, which will improve with the application of adaptive filtering.

## 2. Materials and Methods

### 2.1. Generation of Synthetic Data

The exploration and evaluation of adaptive filters will be performed using both synthetic and real data. The need for using synthetic signals can be explained as follows:

- In real-life signals, for non-invasive acquisition systems, the Alv waves are substantially contaminated with MR-EMG interference. Therefore, it is not possible, in this case, to have the pure Alv waves, unless invasive electrodes are placed directly on the uterine muscle. However, this procedure may raise ethical concerns in most situations and, therefore, it is not practical.
- The aforementioned fact prevents an accurate filter and parameters optimization, regarding MR-EMG attenuation.
- Hence, the need to use analytically defined signals for the MR-EMG and Alv waves, which can only be achieved using synthetic data.
- These synthetic signals should be generated using features and properties as close as possible to the corresponding real data.

Concerning the breathing signal pattern, it is known that the respiratory signal depends on the physiological and pathological parameters of each subject and their physical activity at the time of the EHG signal acquisition (talking, chewing and swallowing, coughing, normal sleep, and sleep with apnea, amongst others). Thus, in this work, three situations were simulated, all with the respiratory signal covering the entire frequency band that is established as being typical of the majority of the population: 0.2 to 0.34 Hz [15]. Three respiratory signals were simulated:

- Smooth (chirp) breathing variation: a linear variation between temporal and frequency limits, from slow to fast breathing. The study for this case will be designated as the first simulation.
- Breathing variation in a triangular wave shape from smooth to fast in 250 seconds for eight cycles. The study for this case will be referred to as the second simulation.
- Random variation of the breathing regime in the previously mentioned band. The study for this case will be referred to as the third simulation.

The Alv waves atoms will be simulated according to the results presented in [9]. The parameters for these cases will be based on the central frequency (f_atom), fractional bandwidth (fract_bw), and fractional bandwidth reference level (fract_bw_r), which adopts the default value of −6 dB and pulse envelope level (pulse_level) corresponding to −60 dB. The −60 dB for the pulse envelope level was chosen because it allows for a time duration that approximates the empirical values.

The central time, which is the location of the Alv atoms in the EHG signal, was selected to achieve overlap with the respiration signal. This corresponds to the worst-case scenario for testing the adaptive filters.

**Figure 1:**
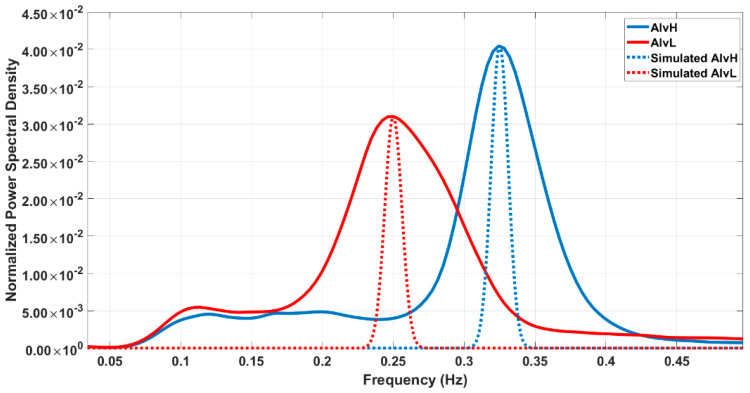
Normalized power spectral density for the average AlvH (blue) and AlvL (red) and their simulated versions in dotted blue and red, respectively. Bandwidth in the simulation has been reduced to distinguish the spectral peaks in the frequency marginals.

Figure 1 shows the normalized power spectral density for the AlvL and AlvH (solid line) and their simulated versions (dotted line). The bandwidth has been reduced to separate the multiple Alv contributions in the frequency marginals. This subject will be explained further ahead.

The simulated Alv and respiratory signals were added up. A white noise component was also included.

**Figure 2:**
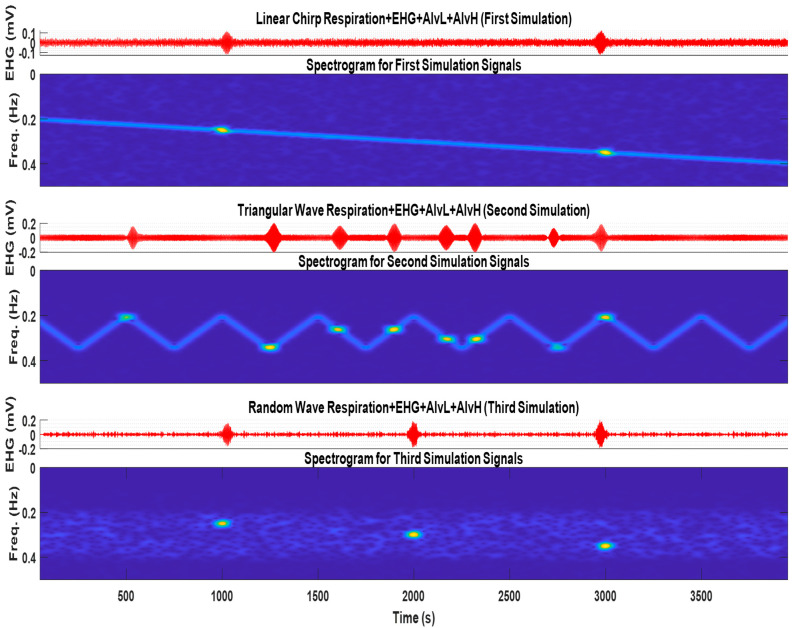
The three synthetic EHG signals, which include the Alv waves and respiratory signal for the chirp respiration, two Alv waves (first and second subfigures), i.e., triangular wave respiration and eight Alv waves (third and fourth subfigures), and random wave respiration and three Alv waves (fifth and sixth subfigures). The spectrograms can represent in time and frequency domain the signal features. For the Alv waves parameters, refer to Table 2.

Figure 2 represents the obtained signals and respective spectrograms for the three simulated cases. In the chirp and triangular respiration cases, it is possible to observe a time and frequency overlap between this signal and Alv atoms. This is considered to challenge the adaptive filters, regarding attenuating respiration and keeping the atoms.

### 2.2. Adaptive Filters and Parameters Optimization and Selection

**Figure 3:**
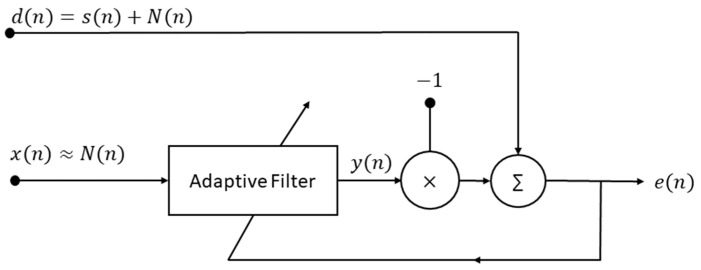
Block diagram for an adaptive filter working as a noise reduction system. Adapted from [47].

For non-stationary signals, whose instantaneous frequency and bandwidth vary in time, classical filters may not be the optimal solution, since the filter parameters are set a priori. In this case, the use of a digital filter capable of readjusting its parameters as time progresses, according to the characteristics of the input signal, is a better solution. These filters are called adaptive filters. One important application of adaptive filters is noise control, which is the subject of the herein presented work. As seen in Figure 3, the input signal is often designated as the desired signal *d*(*n*), and it contains the clean signal *s*(*n*) added to the noise component *N*(*n*). An estimation of the interference signal, *x*(*n*), which should ideally be similar to *N*(*n*), must be present. The value *n* is the discrete sample. The adaptive filter block output *y*(*n*) is an estimation of *N*(*n*), which will be, in the next block, subtracted from the desired signal to produce the *e*(*n*) output, also called the error signal [47]. The subtractor block is what makes the adaptive filter special, since the removal of the offending interference is performed through a subtraction operation, in contrast with the classical filters’ convolutional algorithms.

The following adaptive filters have been tested: least mean squares (LMS), normalized least mean squares (NLMS), frequency domain adaptive filter (FDAF), block least mean squares (BLMS), filtered-X least mean square (FXLMS), sign-error least mean squares (SELMS), sign–data least mean squares (SDLMS), sign–sign least mean squares (SSLMS), least-squares lattice (LSL), fast transversal filter (FTF), recursive least squares (RLS), householder recursive least squares (HRLS), sliding-window recursive least squares (SWRLS), householder sliding-window recursive least squares (HSWRLS), QR-decomposition recursive least squares (QRD-RLS), and Wiener. The selection criterium was to be as comprehensive as possible, considering the computational platform and time planning. Additionally, the code availability and confidence for the selected filters were taken into consideration, and the adaptive filters found in the literature survey for biomedical applications were also considered whenever possible.

For each adaptive filter, a set of parameters should be selected. Often, the authors do not justify their selection criteria. Additionally, frequently, the used parameters’ justification is presented as an inheritance from previous similar studies. In the latter case, there is a risk of non-optimal parameter propagation. Additionally, parameters used for a particular biomedical signal may not be optimized for another one or, indeed, a different framework. In this study, an effort was made to obtain the optimal parameters for all the sixteen tested adaptive filters applied for the maternal respiration interference reduction in the Alv waves contained in the EHG. Additionally, the best performer adaptive filters are quantitatively identified amongst the sixteen candidates.

The used metric was the root mean square error (RMSE) between the EHG, with original simulated Alv waves (without noise component), and output of the filter, where the noise respiration signal (MR-EMG) was the chirp, triangular, or random waveshape, as seen in Figure 2.

**Figure 4:**
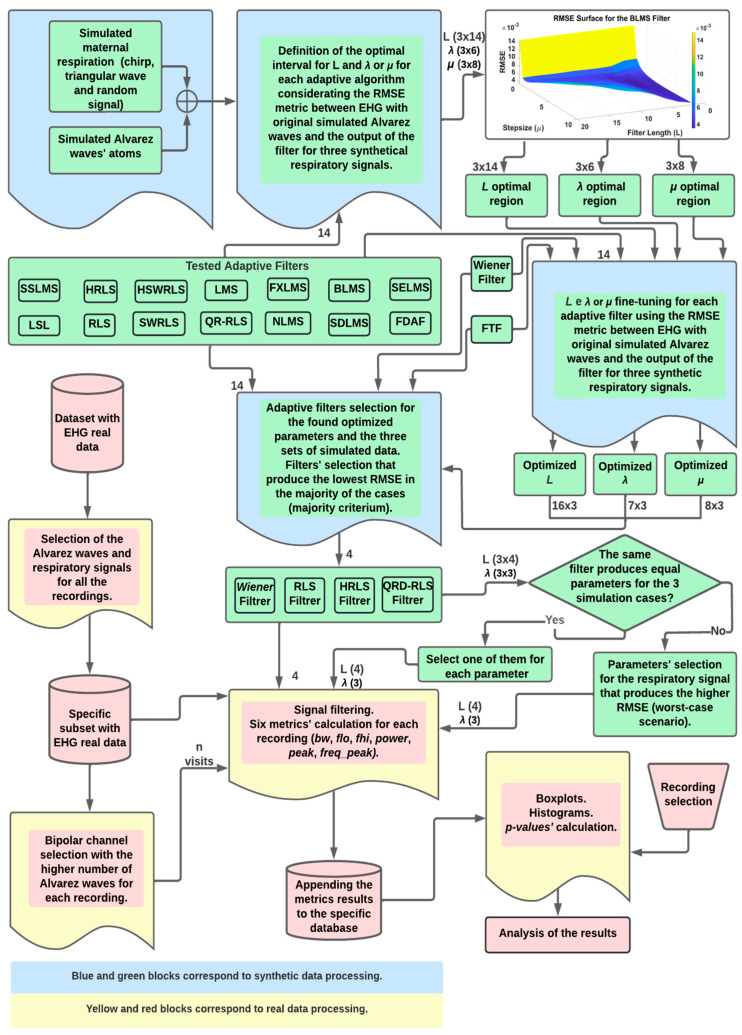
Flowchart for the methodology to obtain the optimized adaptive filters and their parameters for the synthetic EHG data (blue and green blocks). The optimal filters with the obtained optimal parameters are then applied to real EHG data (yellow and red blocks). The results were statistically analyzed.

**Figure 5:**
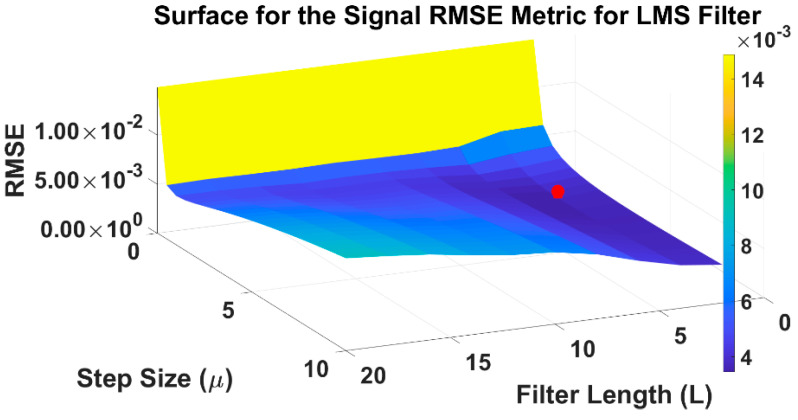
Method for the optimal region coarse determination for the L and μ LMS filter values. These results were obtained for the third simulation signal. The red dot represents the center of the optimal region corresponding to the minimum RMSE. For further information, please see the text.

The flowchart depicting the overall methodology in this work is presented in Figure 4, where blue and green blocks correspond to the synthetic data analysis; yellow and red refer to the real data application. In the top blocks, and after the simulated data generator, the optimal regions for L, μ, and λ are obtained for the fourteen adaptive filters. The best exploratory region is the first step for a coarse determination of the optimal parameters. The operation for this involves finding the minimum value in the tridimensional surface representing the RMSE variation, due to filter parameters’ different values. Figure 5 shows this operation, as performed for the LMS filter. The coarse determination of the optimal region, for the L and μ LMS filter values, for the third simulation signal, is represented by the red pin. In the flowchart, for each filter and each simulated signal, a tridimensional surface is obtained. In total, 42 surfaces are accounted for. The computational burden of this operation is significant, and this requires widening the sampling intervals of the parameters under testing. This strategy leads to the need for a second step for the fine-tuning of the mentioned parameters. Overall, this two-step strategy allows for substantial savings in the computational execution time. This approach was applied to the sixteen adaptive filters and three simulated signals. The fine-tuning operation was performed by finding the minimum RMSE, using a reduced interval in the tested parameters, which allows for testing a higher number of combinations, without incurring excess computational execution time.

The next step is the selection of the best-performing adaptive filters amongst the sixteen studied options. The best-performing adaptive filters will produce the lower RMSE in the majority of the three simulation cases (majority criteria). It turns out that the best performer was the Wiener filter. Given that the real-time implementation of this filter may be, in certain applications, a challenging task, it was decided to score the second-best filter. In this study, three filters break even in second place: RLS, HRLS, and QRD-RLS. In the following step, two outcomes are possible:

- For each filter, if one of the parameters is equally valued in the three simulated cases, it will be selected.
- For each filter, one of the parameters is different for at least two simulation cases. In this case, the parameter corresponding to the second simulation is selected. This corresponds to the worst-case scenario, since this simulated signal contains abrupt changes that are challenging for the adaptive filters.

At this point, the description of the algorithms involving simulated data in the flowchart in Figure 4 is complete.

### 2.3. Application of the Selected Filters to Real Data

For the real data, the Iceland Uterine Electromyography database [48] was used. It comprises 123 EHG recordings from 45 pregnant subjects. The EHG was collected non-invasively from the abdominal surface, with a sampling frequency of 200 Hz. Sixteen (4 × 4) acquisition channels were used. The gestational age at delivery was 39.76 ± 1.40 weeks (mean ± standard deviation).

**Figure 6:**
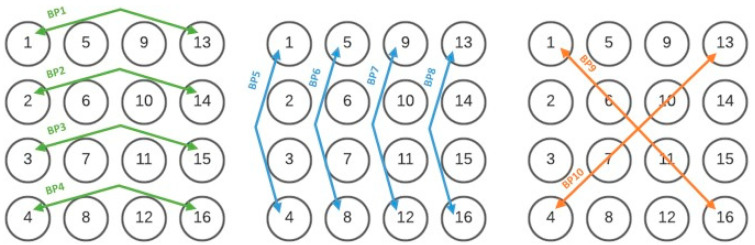
Electrodes arrangement for the Icelandic EHG database. Circles represent monopolar electrodes, and the green, blue, and orange arrows represent the ten bipolar (BP) arrangements selected in [50].

Alv waves dataset from previous work was available, for which they have been automatically detected in the EHG [17]. These components were identified in bipolar channels, shown in Figure 6, where the 4 × 4 Icelandic database electrode structure was represented.

After selecting the four best performance adaptive filters for the reduction of the respiratory signal in the EHG and, consequently, in the Alv waves, as has been explained in previous sections, the next step was to apply these selected filters to the Icelandic EHG data.

It should be emphasized that this step of the work was not about validating the previously executed filter selection or the parameters. As mentioned before, that operation should be performed with simulated data, where the pure and noise signal components are known beforehand, which is only possible with synthetic data.

In this section, the effect of the selected adaptive filters in real data will be explored, namely in the Alv waves. The following features will be included in the study, regarding comparing the periodograms of filtered Alv waves with the non-filtered:

- Bandwidth (*bw*) defined as the frequency band that contains 75% of the energy of each Alv wave, Hz.
- Bandwidth low frequency bound (*flo*), Hz.
- Bandwidth high frequency bound (*fhi*), Hz.
- Signal power in the occupied bandwidth (*power*), Watt.
- Amplitude peak (*peak*), V.
- The frequency corresponding to the amplitude peak (*freq_peak*), Hz.

**Figure 7:**
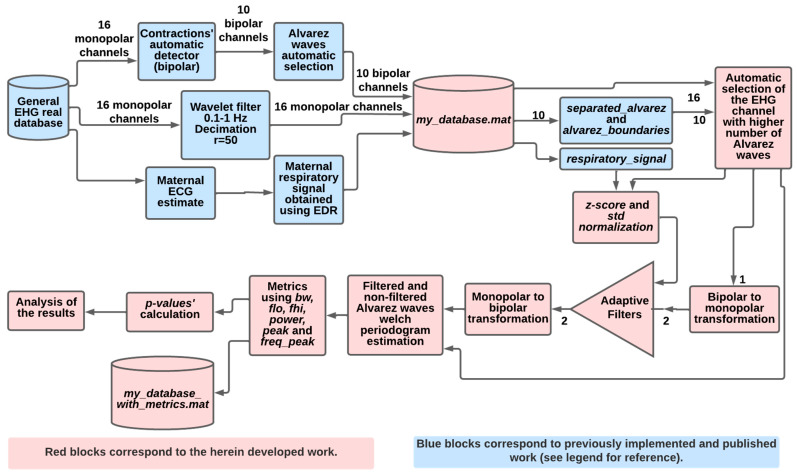
Flowchart of the optimally selected adaptive filters’ application to real EHG data. Blue blocks correspond to previously developed and published work [17]. For further information, please see the text.

The rationale for using the Welch periodogram for Alv waves analysis is described in [17]. The flowchart in Figure 7 highlights the methodology in this section.

For the real data, the respiration signal was obtained through the application of the EDR method to the maternal ECG [49], followed by a post-processing step that includes outlier removal and wavelet filtering between 0.20–0.34 Hz. The used EDR technique produced a signal that was an interpolated time series of the QRS areas in maternal ECG. This requires normalization and rescaling to realistic interference levels. A z-core normalization was applied in the first step, followed by a rescaling using the EHG standard deviation and a division by four. The maternal ECG data was retrieved from the current EHG electrodes. This signal was used as the estimated interference to be input to the adaptive filters. A significant fact here is that the estimation was not obtained from an extra sensor.

In this work, for each recording, the bipolar channel with a higher number of Alv waves was selected to improve statistical validation. Since it has been reported that bipolarity can reduce the respiratory component in the EHG [11], a decision was made to apply the adaptive filters to the monopolar channels arising from the respective bipolar arrangement. This was followed by returning the filtered monopolar channels to the corresponding bipolar arrangement. This last step was mandatory, since the original Alv waves were detected in bipolar channels.

#### Statistical Validation Method

In the following step, statistical validation was performed, regarding the above features, to understand how the optimal adaptive filters modify the original Alv waves. For each feature, the mean value of the raw signal was compared with its filtered version by performing the following two bilateral tests:

$$\;H_{0}^{1}:\mu_{Feature_{i}\left(raw\;signal\right)}=\mu_{Feature_{i}\left(Wiener\;filter\right)}\;\;vs.\;H_{1}^{1}:\mu_{Feature_{i}\left(raw\;signal\right)}\neq \mu_{Feature_{i}\left(Wiener\;filter\right)}$$

$$\;H_{0}^{2}:\mu_{Feature_{i}\left(raw\;signal\right)}=\mu_{Feature_{i}\left(RLS,HRLS,QRD-RLS\right)}\;\;vs.\;H_{1}^{2}:\mu_{Feature_{i}\left(raw\;signal\right)}\neq \mu_{Feature_{i}\left(RLS,HRLS,QRD-RLS\right)}$$

where

- $\mu_{Feature_{i}\left(raw\;signal\right)}$ represents the mean value of the raw signal,
- $\mu_{Feature_{i}\left(Wiener\;filter\right)}$ is the mean value of the outputs of the Wiener filter,
- $\mu_{Feature_{i}\left(RLS,HRLS,QRD-RLS\right)}$ is the mean value of the output of RLS/HRLS/QRD-RLS filters, for the *i-th* feature (*i* = 1, …, 6).

The superscripts in the null and alternative hypotheses $H_{0}^{j}$, $H_{1}^{j}$, *j* = 1,2, distinguish between the comparison of the raw signal with the Wiener and RLS/HRLS/ QRD-RLS filtered signals, respectively.

To carry out the above hypotheses testing, paired z-tests were performed; therefore, no distributional assumptions were made to the underlying populations [51]. The *p*-values were adjusted by the Bonferroni procedure, since there are two hypotheses for each feature. Let $p_{i}^{j}$, *j* = 1,2 denote the unadjusted *p*-value of the *j-th* hypothesis testing for the *i-th* feature; then, the Bonferroni adjusted *p*-value is given by $p_{Bonf,\;i}^{j}=\min\left\{1,kp_{i}^{j}\right\}$, *j* = 1,2, where *k* is the number of hypotheses under study, which, in this work, is *k* = 2 [52,53].

## 3. Results

### 3.1. Application to Synthetic Data

In the first run, the boundaries and steps for the filter parameters were established empirically, with the following constraint: A minimum would have to be present in RMSE values of the surface plot, whose independent variables are the parameters, as seen in Figure 5 and Section 2.2. Table 3 summarizes the initial values that will be the seed for the remaining algorithms.

The next step is to input these seed values to build a three-dimensional plot, where the interest optimal region was defined as the area around the minimum in the surface plot. This minimum corresponded to the lowest RMSE value on this surface. This is illustrated in the first line of the flowchart in Figure 4. However, the Wiener filter was not coarse optimized, since it depends on one parameter only, L. For the FTF filter, it is recommended that the λ value be within the [1–0.5/L, 1] interval, so the coarse optimization was also skipped.

**Figure 8:**
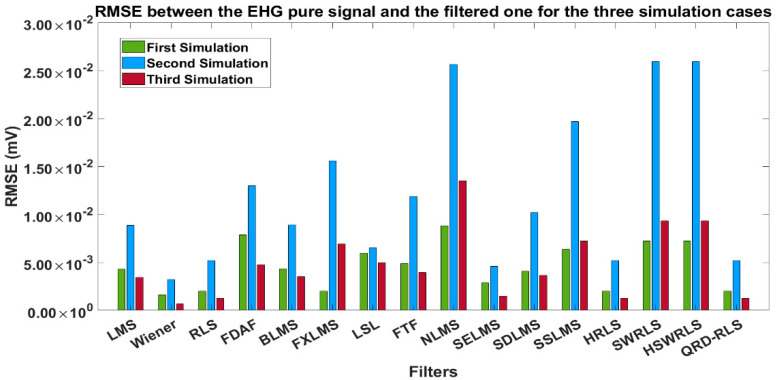
Representation of RMSE values in Table 5.

Fine-tuning was then applied to the interest region to obtain optimal values for L, μ, and λ for each filter and simulation type. Table 4 summarizes the optimized values for three simulation signals and each adaptive filter. Using the optimized parameters in Table 4, for the three simulation signals and the sixteen filters, the RMSE error was obtained between the pure EHG and filtered one. The results are shown in Table 5, with Figure 8 being the corresponding pictorial representation.

The lowest RMSE in the majority of the simulation cases is the criterium for optimal filter selection. In Table 5 (blue cells correspond to low RMSE values), the following comments apply:

- The lowest RMSE for the three simulation signals corresponded to the Wiener filter. This filter implementation may be challenging for certain real-time applications. Hence, the need to score other filters.
- In the first and third simulations, the lowest RMSE values corresponded to the RLS; HRLS and QRD-RLS tied.
- Since the lowest RMSE value of the second simulation corresponded to the SELMS and, using a majority criterion, the winners after the Wiener filter were: RLS, HRLS, and QRD-RLS alike.

For the Wiener, RLS, HRLS, and QRD-RLS filters, the optimal parameters for the three simulation cases were identical (L = 2 for all of them and λ = 1 for the RLS, HRLS, and QRD-RLS). If the parameters were different, the criterium would be to select those corresponding to the second simulation case, which is considered the worst-case scenario, regarding the respiration signal.

**Figure 9:**
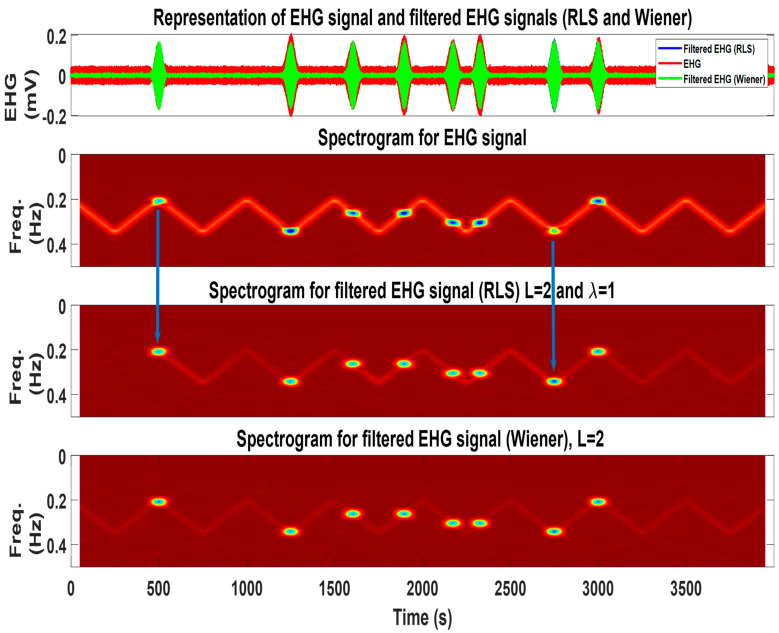
In the first subplot: representation of the EHG signal with eight Alv atoms (red) and filtered output for Wiener (green) and RLS (blue) filters. The filtered Alv atoms are shown in green and blue (practically overlapped). Second subplot: the time-frequency representation of the EHG signal containing the respiration interference and eighth Alv atoms, which are visible and purposedly placed over the respiration signal. Third subplot: output of the RLS filter, where the respiration signal is attenuated and Alv waves are well-preserved. The blue arrows represent two cases, where the amplitude of the filtered Alv atoms increased after filtering (note the darker atoms’ color). This reveals a counter-phase relation between the respiratory signal and Alv waves in the original signal. Fourth subplot: output of the Wiener filter, where a further attenuation of the respiratory component can be observed. This is consistent with the obtained result, whereas the Wiener filter is the best performer.

Figure 9 summarizes the input and output for the RLS and Wiener filters for the second simulation case. For further explanation regarding this figure, please refer to its caption.

**Figure 10:**
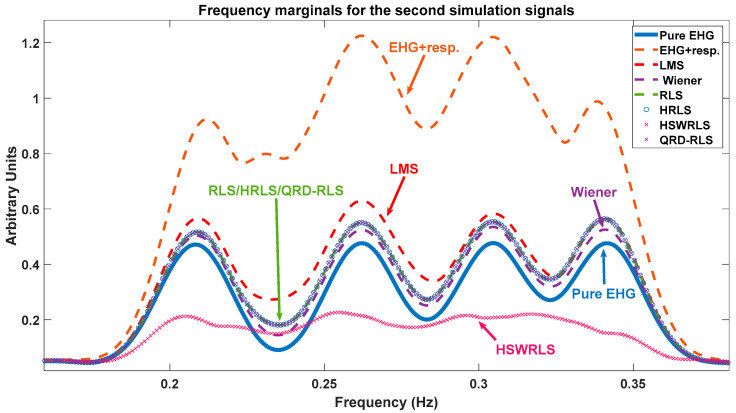
Frequency marginals for the second simulation data. The analysis should be performed, relative to the EHG + resp (red dash) and pure EHG (blue) curves. The best-performing filter output using the marginal criterium should approach the pure EHG curve. In that respect, the best and worst performers are the Wiener and HSWRLS filters, respectively. The RLS/HRLS/QRD-RLS filters are the second-best performers after the Wiener. The LMS filter performs below the RLS/HRLS/QRD-RLS filters.

The frequency marginal, which is represented in Figure 10, is herein used for qualitative analysis only, since a quantitative metric using this data would be negatively affected by the fact that this representation arises from a projection of the signal into the time-frequency domain that may not respect their ideal properties, such as time support and total energy. Further insight regarding the frequency marginals is provided in the caption of Figure 10.

### 3.2. Application to Real Data

**Figure 11:**
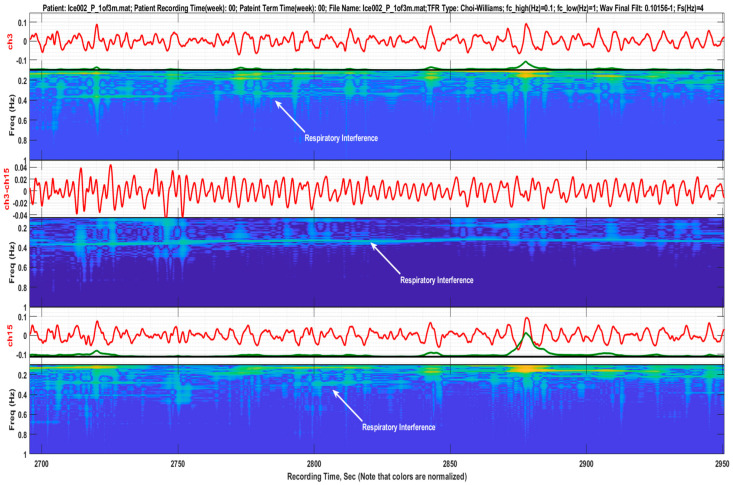
Illustration of how bipolar channels may enhance the respiratory signal, instead of reducing it. This can be explained by counter-phase respiratory signals in each of the monopolar electrodes, possibly generated by certain patterns of respiratory muscle movements.

Using the bipolar montage to reduce respiratory interference should be performed with caution, since the reverse outcome can happen. Figure 11 depicts a case where bipolarity led to the amplitude enhancement of the respiratory interference. This can be explained by the counter-phase respiratory signals in each of the monopolar electrodes, possibly generated by certain patterns of respiratory muscle movements. This behavior has been observed in several EHG recordings of the database in use.

**Figure 12:**
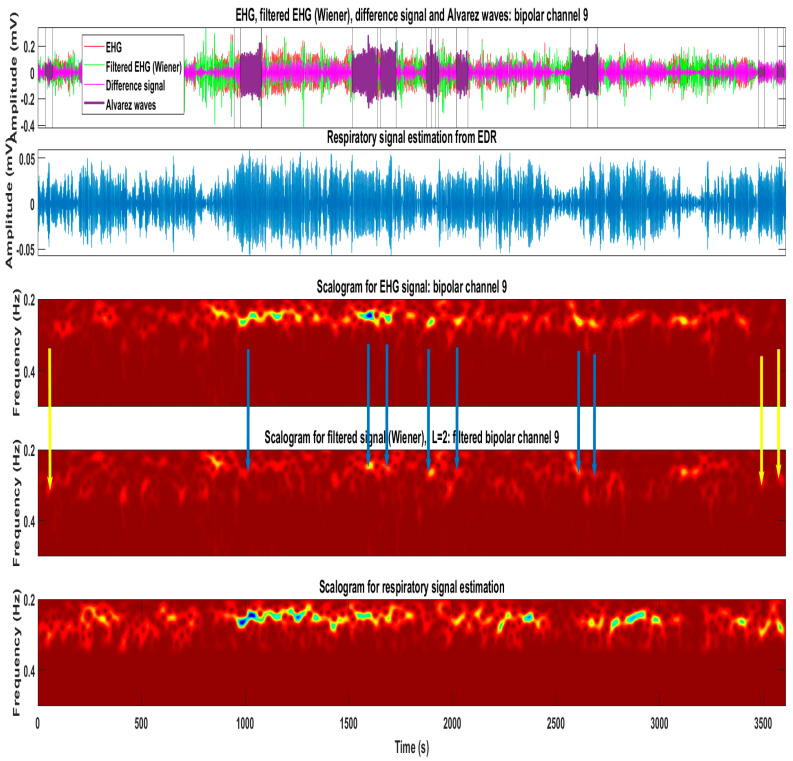
The first subplot represents a real EHG recording, where 75 Alv have been detected. For readability reasons, only 11 Alv waves are represented, in purple. The vertical lines represent their boundaries. The green line represents the Wiener EHG filtered signal, and the pink line is the signal difference. The second subplot represents the EDR estimation respiratory signal. The third subplot represents the scalogram of the EHG signal, where the respiratory component is present, overlapped with Alv waves. In the fourth subplot, which represents the scalogram for the Wiener filter, it was observed that the respiratory signal has been substantially reduced. Regarding Alv waves, two behaviors are present the yellow arrows represent cases where Alv waves amplitude increased after filtering and otherwise (blue arrows). This reveals, in the first case, that the respiratory signal and Alv waves are in a counter-phase, whereas, in the second case, they are in phase. The fifth subplot represents the scalogram for the EDR estimated respiratory signal. The scalograms are obtained using the *cmor-25-1* wavelet [11].

Figure 12 represents an example of the application of the Wiener filter to a real EHG recording retrieved from the used dataset. There are 75 Alv waves in this signal. For readability reasons, only 11 are represented, in purple colour. The time-frequency plots allow for the observation of the filter impact, leading to the attenuation of the MR-EMG. For further details, please refer to the Figure 12 caption.

**Figure 13:**
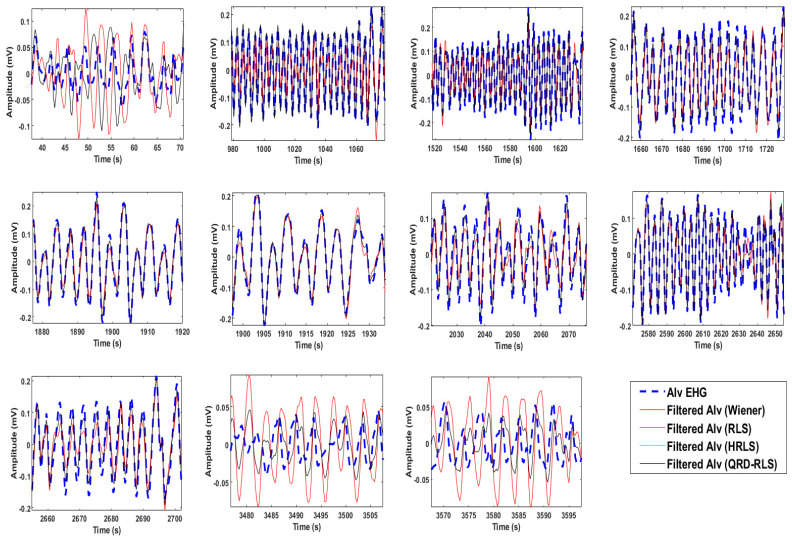
Eleven real-life Alv waves in the time domain (blue dash) from one EHG recording of bipolar channel 9. The filtered versions for the Wiener (red), RLS (pink), HRLS (cyan), and QRD-RLS (black) are shown. The last three are overlapped.

**Figure 14:**
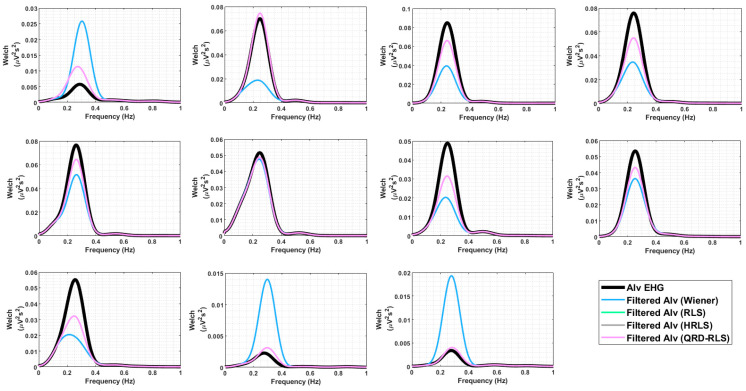
Welch periodograms for the 11 real-life Alv waves presented in Figure 13. Each plot represents the unfiltered Alv waves (black) and filtered ones by Wiener (blue), RLS (green), HRLS (brown), and QRD-RLS (pink) filters. The last three are overlapped.

**Figure 15:**
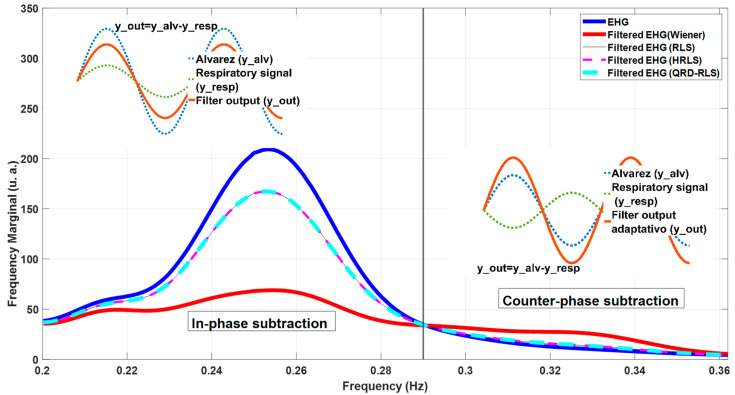
A frequency marginal example for an EHG recording (blue) and filtered EHG: Wiener (red), RLS (brown), HRLS (pink), and QRD-RLS (cyan). The number of Alv waves included in this computation was 75. The last three marginal curves are overlapped. For the Wiener filter (red), it was observed that, for frequencies under 0.29 Hz, the filtered output has an amplitude below the input EHG (blue), whereas, above this frequency, an amplitude increase relative to the blue line is registered. This means that, in the first case, there was an in-phase subtraction between the EHG signal and estimated noise filter output; in the second case, a counter-phase subtraction occurred. The two side plots explain this behaviour using sinusoidal waves.

Figure 13 represents the original Alv waves for the bipolar channel 9 EHG recordings. It is possible to compare the original version of the waves (blue dash) with the output of different filters (see caption for details). The 1st, 10th, and 11th subplots, horizontally-wise, depict the cases where the filtered signal amplitudes are above the original Alv waves. This indicates a counter-phase effect between the Alv waves and MR-EMG. The 3rd, 4th, and 9th subplots represent the reverse situation, indicating that Alv waves and the MR-EMG are in-phase. In Figure 14, the Welch periodograms of the above waves are illustrated, for which the comments presented in Figure 13 apply, regarding the relative amplitudes of these frequency representations. For example, the last two Alv waves (horizontally wise) in Figure 12 and Figure 13 show the following trend: the output of the Wiener filter produces a higher amplitude signal and, consequently, higher amplitude periodogram, compared to the non-filtered Alv. In classical theory, digital filters designed without a gain feature are not, generally, supposed to display this behavior, which is, however, in many instances, observed in this study, from which the above-mentioned Alv waves are just an example. The frequency marginals presented in Figure 15 are a helpful tool for understanding this behavior. Please see the figure caption for more details.

**Figure 16:**
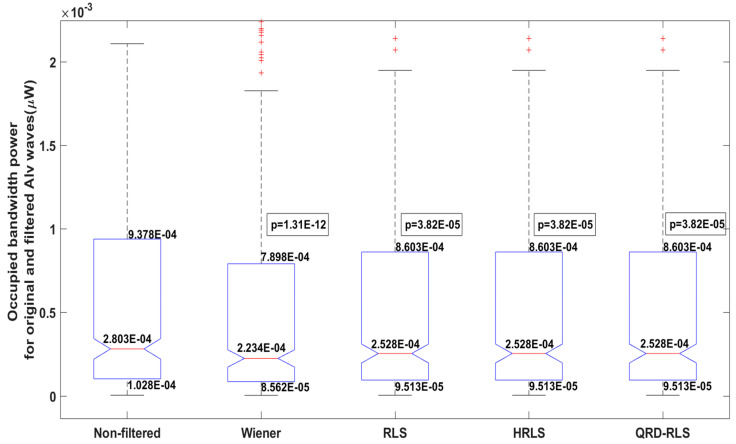
Boxplots for the Alv waves before (non-filtered) and after being filtered by the Wiener, RLS, HRLS, and QRD-RLS filters. The studied metric is the occupied bandwidth power. A decrease in median and quartiles was observed for all the filtered boxplots. This reveals that the adaptive digital filters attenuated the respiratory interference. Additionally, it reveals that interference is mostly in-phase, and the Wiener filter produces a higher attenuation. All the Bonferroni’s adjusted *p*-values, based on paired z-tests, validated this hypothesis.

Figure 16 illustrates the boxplots for the Alv waves before (non-filtered) and after being filtered by the Wiener, RLS, HRLS, and QRD-RLS filters. All the filtered boxplots show a decrease in the power of the filtered periodograms. For details, please see the figure caption. For the other five features, similar boxplots are also available. However, to restrain this document length, a table with the general result is otherwise presented.

Table 6 represents the features’ variation between non-filtered Alv waves and their versions after adaptive filtering for respiration attenuation in real data.

- A substantial power decrease (∆*power*) was observed for the Wiener filter (*p* = 1.31 × 10^−12^). For the other filters (*p* = 3.82 × 10^−5^), the power decrease was roughly halved in the median and quartiles, relative to the Wiener filter. This may reflect the Wiener filter outperforming the attenuation of respiratory signal, as has been verified in the simulation data. This also highlights that the in-phase subtraction between the noise estimation and Alv waves was prevalent, relative to the counter-phase one.
- The bandwidth increase (∆*bw*) for all filters (*p* = 1.20 ×10^−45^ for Wiener and *p* = 1.66 × 10^−12^ for other filters) was a direct consequence of the reduction of the power (∆*power*). To allow for the 75% energy ratio, the bandwidth must increase.
- To allow for the bandwidth increase, the frequency limits ∆*flo* (*p* = 0.00 for Wiener and *p* = 4.44 × 10^−16^ for other filters) and ∆*fhi* (*p* = 2.46 × 10^−28^ for Wiener and *p* = 6.39 × 10^−9^ for other filters) decreased and increased, respectively.
- A substantial decrease in the periodograms peak (∆*peak*) was observed, which is consistent with the above-reported decrease in the power (∆*power*) for the Wiener filter (*p* = 4.11 × 10^−13^). For the other filters, the ∆peak decrease (*p* = 1.46 × 10^−5^) was roughly halved in the median and quartiles, relative to the Wiener filter.
- Since the ∆*flo* decrease was higher than the ∆*fhi* increase, the peak frequency deviation (∆*freq_peak*) was negative or negligible in all cases (*p* = 1.19 × 10^−8^ for Wiener and *p* = 3.59 × 10^−4^ for other filters). This indicates that the attenuation of the respiratory signal led to a lower frequency peak.

## 4. Discussion and Conclusions

The innovative components in this work are as follows:

- Maternal respiratory signal (MR-EMG) attenuation in the EHG, which is a problem often overlooked or dealt with, in many cases, using bipolar channels.
- Algorithm development to find the optimal adaptive filters and their parameters for the MR-EMG attenuation in the EHG.
- A contribution to adaptive filtering behavior insight, which is important for the interpretation of the results. This information could be relevant for other researchers in the biomedical signal processing field.

Maternal respiration is one of the most relevant interferences in the EHG, given that its frequency band (0.2–0.34 Hz) overlaps with important EHG components, such as Alv waves. Typically, bipolar channels are mounted, in an attempt to cancel the common mode MR-EMG in the respective monopolar configuration. However, this operation, as mentioned earlier in this work, can have the exact opposite effect of enhancing the respiratory signal. This can be explained by the counter-phase MR-EMG presence in each monopolar channel from which the bipolar one was obtained. Moreover, bipolarity can attenuate the signal of interest if it is present in both monopolar channels, mainly if they are relatively close by. Classical filtering, designed to reduce the MR-EMG, will also affect the signal of interest in the shared bandwidth, so it is not the ideal solution.

Adaptive filters seem to overcome these predicaments, since, as explained earlier, their operation is based on the subtraction of the estimated interference from the composite signal of interest. In optimal conditions, this behavior allows for the attenuation of the offending signal, which is added to the composite one, without disturbing the bandwidth of the signal of interest. In terms of the work herein presented, the composite signal is the EHG that inevitability contains the offending MR-EMG. Therefore, the application of adaptive filters may preclude the need for the montage of bipolar channels.

An estimation of the MR-EMG, which is necessary as an input to the adaptive filters, is unavailable on the existing open-source EHG databases. In this work, an innovative approach included the estimation of the MR-EMG from the maternal ECG recorded in the EHG electrode locations, using the well-established EDR technique.

An algorithm was developed to obtain the optimal filter and respective parameters for the task at hand. The filters available for selection were Wiener, LMS, NLMS, BLMS, FXLMS, FDAF, SELMS, SDLMS, SSLMS, FTF, LSL, RLS, HRLS, SWRLS, HSWRLS, and QRD-RLS. In the selection step, synthetic data was used. The best performers were Wiener (L = 2), RLS, HRLS, and QRD-RLS (L = 2 and λ = 1). This algorithm can be used for other scenarios, where the users are looking for the optimal adaptive filter and parameters’ solutions for their applications.

As a complementary work component, the obtained optimal filters were applied to real data from the Icelandic Reykjavik University EHG database. This step was not meant to be for the filter and parameters validation, but rather a study regarding the effect of the optimal filters on the Alv waves, before and after MR-EMG attenuation. A set of Alv waves features were selected for analysis. It was found that, in all cases, the respiration signal was attenuated from the original Alv waves, which led to an increase in bandwidth and decrease in their power content. In practical setups of algorithm development using Alv waves, it would make sense to apply one of the selected filters to the EHG as a first pre-processing step.

## Figures and Tables

**Table 1:** State of the art regarding adaptive filters applied to the EHG.

Authors and Year	Purpose of the Work	Reference Signal	Filters	Parameters and Data	Results and Observations
Jérémy Terrien et al., 2007 [19]	Exploring the performance of RLS and LMS adaptive filters as a preprocessing technique for monopolar EHG signals. The used metric was the signal-to-noise ratio.	Average of the four vertical EHG monopolar channels	RLS and LMS (adaptive) and Laplacian (non-adaptive)	fs = 200 Hz; ε = 0.001; λ = 1; μ = 0.1; L = (4,8,12,16,20,24,28,32,36,40,48,56). Measurements were performed by a 16-channel physiological signal recorder. The sample was from two women in spontaneous labour (37 and 39 weeks).	Contractions’ segmentation was performed manually. The RLS filter provides a higher signal-to-noise ratio than the LMS filter. The RLS filter is less sensitive to the filter order than the LMS filter. The highest signal-to-noise ratio was obtained for an RLS filter of order 4. For the LMS filter, the signal-to-noise ratio gets worse as the filter order increases. Statistical analysis: there are no differences between the results obtained by the RLS and Laplacian filters. Qualitative analysis: the RLS algorithm results have a higher signal-to-noise ratio than the Laplacian filter. RLS adaptive filtering shows the best results when applied to the monopolar EHG.
Shi-jin Liu et al., 2011 [36]	Maternal ECG signal attenuation, followed by fECG extraction using adaptive filtering algorithms (RLS and NLMS).	Maternal ECG	RLS and NLMS (adaptive)	fs = 500 Hz; λ = 0.98; μ = 0.16; L = 4; σ = 0.001. The used data corresponds to real signals. They were recorded by placing skin electrodes on the abdominal region. Each signal contains 4000 sampling points.	The RLS algorithm is more robust than the NLMS method and converges faster. The RLS algorithm is one of the signal processing techniques suitable for fECG extraction.
Jamila Khalaf et al., 2013 [37]	Directly retrieves the fECG from the composite signals, without using the maternal ECG reference signal.	ICA estimation of the maternal ECG	LMS (adaptive) and bandpass filter between 1 and 150 Hz (non-adaptive)	fs = 1 kHz. Parameters’ information was undisclosed. Clinical data pool: abdominal surface recordings from five women between 38 to 41 weeks of gestation, collected during labour. Each data set comprises six different signals: four composite signals through four electrodes. A direct fECG recorded from the fetal scalp was used for correlation analysis.	All recordings were bandpass filtered between 1 and 150 Hz and then normalized by the standard deviation. The ICA technique was used. It was possible to attenuate the maternal ECG signal to obtain only the fECG by combining a blind source separation technique with adaptive filtering.
Ales Smrdel et al., 2015 [40]	Prediction and classification of EHG term and pre-term recordings.	Undisclosed	RLS (adaptive)	fs = 20 Hz; λ = 0.9995; L = 12. Several parameters were tested, and the best performer was presented. Data pool: term–preterm EHG (TP-EHG) database (300 recordings).	A sample entropy technique was additionally used. In the power spectra of the signals that correspond to term births, the peaks appear in the frequency band up to 1 Hz. Premature birth recordings show spectral peaks that are less prominent and appear below 1 Hz Both recordings’ types present peaks around the frequencies of 3 and 4 Hz.
Manel Limem et al., 2016 [20]	Attenuation of the electronic electromagnetic and ECG maternal interferences present in the EHG, using the adaptive filter algorithms. Comparing the results of the adaptive filtering methods with the wavelet transform and bandpass filter.	The uterine electromyography signal was combined with random noise	LMS and RLS (adaptive) and bandpass filter between 0.34 and 1 Hz and discrete wavelet transform: db4 (non-adaptive)	Filter parameters undisclosed. Four EHG signals from the TP-EHG database were used.	The RLS filter provides better results regarding noise attenuation in uterine electromyography. RLS and LMS filters were compared with bandpass filtering and wavelet transform methods through the values of the signal-to-noise ratio. The RLS algorithm provides a better signal-to-noise ratio.
Kahankova et al., 2017 [38]	Extracting the fECG from the EHG signal using some finite impulse response (FIR) adaptive filters, based on the RLS algorithm. Evaluation of the filter’s performance.	Maternal ECG	RLS and FTF (adaptive)	RLS (λ = 1; L = 30); FTF (L = 15; δ = 0.01). These parameters were optimized for the used synthetic data.	RLS algorithm outperforms the FTF. The FTF algorithm is unstable and, therefore, not suitable for extracting the fECG. The RLS filter is a more robust algorithm for the application. A grid of parameters was tested for optimization.

fs: sampling frequency; ε: smoothing factor; λ: forgetting factor; μ: step size; L: filter length; σ: low positive constant; δ: soft constraint initialization factor.

**Table 2:** Parameters for the simulated Alv waves.

Simulation Type	Bandwidth (Hz)	Central Frequency (Hz)	Central Time (s)
First simulation	0.02	0.2500	1000
		0.3500	3000
Second simulation	0.02	0.2084	500
		0.3415	1250
		0.2622	1604
		0.2622	1896
		0.3043	2173
		0.3043	2327
		0.3415	2750
		0.2084	3000
Third simulation	0.02	0.2500	1000
		0.3000	2000
		0.3500	3000

**Table 3:** Parameters’ limits and steps that ensured an RMSE minimum in the surface plot for the second simulation data.

	L			μ			λ		
Filter	Min	Max	Step	Min	Max	Step	Min	Max	Step
LMS	2	20	2	0.0	10.0	0.5	-	-	-
NLMS	2	20	2	0.0+	2.0	0.5	-	-	-
FDAF	2	20	2	0.0+	1.0	0.2	-	-	-
BLMS	2	20	2	0.0	10.0	0.5	-	-	-
FXLMS	2	20	2	0.0	10.0	0.5	-	-	-
SELMS	2	20	2	0.0	10.0	0.5	-	-	-
SDLMS	2	20	2	0.0	3.0	0.5	-	-	-
SSLMS	2	20	2	0.0	5.0	0.5	-	-	-
LSL	5	100	5	-	-	-	0.10	1.00	0.10
RLS	2	20	2	-	-	-	0.95	1.00	0.01
HRLS	2	20	2	-	-	-	0.40	1.00	0.10
SWRLS	2	20	2	-	-	-	0.95	1.00	0.01
HSWRLS	2	20	2	-	-	-	0.20	1.00	0.10
QRD-RLS	2	20	2	-	-	-	0.10	1.00	0.10

**Table 4:** Optimized (after fine-tuning) parameters for the three simulation cases.

Filter	Sim. Type	L	μ	λ	Filter	Sim. Type	L	μ	λ
LMS	1st Sim.	2	4.80	-	FTF	1st Sim.	2	-	0.99
	2nd Sim.	2	0.70			2nd Sim.	11		1.00
	3rd Sim.	4	3.70			3rd Sim.	2		0.98
NLMS	1st Sim.	11	0.05	-	LSL	1st Sim.	6	-	1.00
	2nd Sim.	2	0.05			2nd Sim.	3		1.00
	3rd Sim.	4	0.05			3rd Sim.	33		1.00
FDAF	1st Sim.	16	0.10	-	RLS	1st Sim.	2	-	1.00
	2nd Sim.	10	0.10			2nd Sim.	2		1.00
	3rd Sim.	13	0.10			3rd Sim.	2		1.00
BLMS	1st Sim.	2	4.60	-	HRLS	1st Sim.	2	-	1.00
	2nd Sim.	2	0.70			2nd Sim.	2		1.00
	3rd Sim.	4	3.50			3rd Sim.	2		1.00
FXLMS	1st Sim.	7	1.70	-	SWRLS	1st Sim.	2	-	1.00
	2nd Sim.	6	0.20			2nd Sim.	2		1.00
	3rd Sim.	10	2.50			3rd Sim.	2		1.00
SELMS	1st Sim.	2	0.15	-	HSWRLS	1st Sim.	2	-	1.00
	2nd Sim.	2	0.05			2nd Sim.	2		1.00
	3rd Sim.	2	0.20			3rd Sim.	2		1.00
SDLMS	1st Sim.	2	0.10	-	QRD-RLS	1st Sim.	2	-	1.00
	2nd Sim.	2	0.05			2nd Sim.	2		1.00
	3rd Sim.	4	0.05			3rd Sim.	2		1.00
SSLMS	1st Sim.	2	0.05	-	Wiener	1st Sim.	2	-	-
	2nd Sim.	2	0.05			2nd Sim.	2		
	3rd Sim.	2	0.05			3rd Sim.	2		

**Table 5:** RMSE between EHG pure signal and filtered one for the three simulation cases. The blue cells correspond to the best-performer filters/parameters in each simulation.

Filters	First Simulation Signals	Second Simulation Signals	Third Simulation Signals
LMS	4. 29 × 10^−3^	8. 85 × 10^−3^	3. 44 × 10^−3^
NLMS	8. 80 × 10^−3^	2. 56 × 10^−2^	1. 35 × 10^−2^
FDAF	7. 85 × 10^−3^	1. 30 × 10^−2^	4. 78 × 10^−3^
BLMS	4. 34 × 10^−3^	8. 90 × 10^−3^	3. 52 × 10^−3^
FXLMS	5. 21 × 10^−3^	1. 56 × 10^−2^	6. 92 × 10^−3^
SELMS	2. 81 × 10^−3^	4. 60 × 10^−3^	1. 49 × 10^−3^
SDLMS	4. 09 × 10^−3^	1. 02 × 10^−2^	3. 66 × 10^−3^
SSLMS	6. 35 × 10^−3^	1. 97 × 10^−2^	7. 24 × 10^−3^
FTF	4. 89 × 10^−3^	1. 19 × 10^−2^	3. 95 × 10^−3^
LSL	5. 96 × 10^−3^	6. 55 × 10^−3^	5. 00 × 10^−3^
RLS	2. 01 × 10^−3^	5. 18 × 10^−3^	1. 25 × 10^−3^
HRLS	2. 01 × 10^−3^	5. 18 × 10^−3^	1. 25 × 10^−3^
SWRLS	7. 21 × 10^−3^	2. 59 × 10^−2^	9. 31 × 10^−3^
HSWRLS	7. 21 × 10^−3^	2. 59 × 10^−2^	9. 31 × 10^−3^
QRD-RLS	2. 01 × 10^−3^	5. 18 × 10^−3^	1. 25 × 10^−3^
Wiener	1. 60 × 10^−3^	3. 21 × 10^−3^	6. 87 × 10^−4^
**Mean Error**	4. 79 × 10^−3^	1. 22 × 10^−2^	4. 79 × 10^−3^

**Table 6:** Features’ variation between non-filtered Alv waves and their versions after adaptive filtering for respiration attenuation in real data.

Filters	Statistical Parameters	Features Variation Relative to the Non-Filtered Alv					
		∆*bw* (%)	∆*flo* (%)	∆*fhi* (%)	∆*power* (%)	∆*peak* (%)	∆*freq_peak* (%)
**Wiener**	**Quartile 1**	10.88	−7.82	4.71	−16.74	−28.96	−4.84
	**Median**	11.18	−7.09	3.63	−20.32	−25.39	−2.94
	**Quartile 3**	11.40	−5.52	3.86	−15.78	−18.64	0.00
	**Mean**	11.42	−6.54	4.03	−26.82	−35.45	−2.94
	***p-value***	1.20 × 10^−45^	0.00	2.46 × 10^−28^	1.31 × 10^−12^	4.11 × 10^−13^	1.19 × 10^−8^
**RLS** **HRLS** **QRD-RLS**	**Quartile 1**	2.87	−4.34	1.83	−7.48	−13.74	−3.22
	**Median**	4.35	−2.41	1.83	−9.81	−14.77	−1.47
	**Quartile 3**	5.31	−1.95	1.62	−8.26	−7.11	0.00
	**Mean**	5.02	−2.61	1.61	−8.50	−11.43	−1.10
	***p-value***	1.66 × 10^−12^	4.44 × 10^−16^	6.39 × 10^−9^	3.82 × 10^−5^	1.46 × 10^−5^	3.59 × 10^−4^

## Data Availability

The dataset used in this work is open access: https://physionet.org/content/ehgdb/1.0.0/ (accessed on 30 October 2021).
